# Online sexual violence prevention on a female college campus in India: Evaluation of the RISE-ON program

**DOI:** 10.1016/j.ijchp.2024.100470

**Published:** 2024-05-21

**Authors:** Christina Nieder, Kim Thomae, Joscha Kärtner

**Affiliations:** a University of Münster, Germany; b Christoph-Dornier-Clinic for Psychotherapy, Münster, Germany

**Keywords:** Sexual violence, Sexual assault, Prevention, Online Programs, India

## Abstract

**Background:**

Sexual violence represents a severe problem for young Indian women and requires effective prevention. Since face-to-face prevention programs are limited in reach, we developed the online sexual violence prevention program RISE-ON consisting of three modules, namely Gender, Sexual Violence, and Bystander Education. The study's objective is to investigate the short-term effects of the RISE-ON modules on participants’ knowledge and attitudes.

**Method:**

A total of *N* = 244 female college students from Delhi aged 17 to 22 were randomly assigned to 1 of 3 groups with two of the three modules. By design, each group functions as a treatment group for the two included modules and as a control group for the third, missing module.

**Results:**

From pre- to posttest, there were significantly larger increases of participants’ knowledge on gender, sexual violence, and bystander education in the treatment than in the control group. Concerning attitudes, we found significant increases for gender awareness and bystander attitudes across all groups.

**Conclusions:**

This study demonstrates that the RISE-ON modules are effective in terms of increasing knowledge, but there were no module-specific changes of attitudes. Thus, future online prevention programs need to focus increasingly on attitudes, especially attitudes about sexual violence, and behavior change.

## Introduction

Sexual violence is a severe problem affecting millions of people worldwide, whereby women experience violence more often than men (Conley et al., 2017). Sexual victimization not only increases the chances of addictive and sexually risky behaviors (Kaufman et al., 2019; Pengpid & Peltzer, 2020), but it may also cause further mental health problems including post-traumatic stress disorder and suicidal ideations (Chang et al., 2020; Kumar et al., 2013). Based on the definition by Basile et al. (2014), our understanding of sexual violence ranges from rape (i.e., non-consensual sexual acts) to attempted rape or unwanted touching (i.e., abusive sexual contact) and sexual harassment (i.e., noncontact sexual abuse). Though women of all ages face sexual violence, the risk of sexual violence is higher for young women, such as college students (Kennedy et al., 2018). Research in the US revealed that at least 1 in 5 women experienced sexual violence (i.e., rape, attempted rape, and unwanted sexual advances) while in college (Conley et al., 2017).

Similarly, in India, recent studies confirmed a high prevalence of sexual violence against young women. Thus, in the past year 1 in 10 Indian women (aged 15 to 24) fell victim to sexual violence (i.e., primarily rape) by an intimate partner (Ler et al., 2020), and nearly all young women have experienced sexual harassment (Akhtar, 2013; Dhillon & Bakaya, 2014), but female college students were one of the most vulnerable groups (among women) regarding public sexual harassment (Tripathi, Borrion, & Belur, 2017; Valan, 2020). In an interview study, female students in Delhi further expressed an enormous fear of falling victim to sexual violence, resulting in restrictions as they went about their daily life (Nieder et al., 2019). Factors contributing to the precarious situation of female students in India are persisting patriarchal values and traditional gender roles in society (Hunnicutt, 2009; Weitzman, 2014), a lack of proper sex education (Brahme et al., 2020) and a high acceptance of rape myths (i.e., false attitudes and beliefs about rape) among college students (Qureshi et al., 2020).

To tackle sexual violence, researchers agree that effective prevention measures must be put in place to address issues like gender, sexual violence, and bystander education (Foshee et al., 2005; Taylor et al., 2013). A recently developed culture-sensitive face-to-face sexual violence prevention program for female college students in India (RISE) considered these three aspects and showed promising effects. In this program, participants showed an increase of knowledge (i.e., on gender, sexual violence, and bystander education) and a positive change of attitudes (i.e., an increasing awareness of gender stereotypes, bystander efficacy, and intentions to intervene; Nieder et al., 2020). But, despite their proven efficacy, face-to-face prevention programs entail several disadvantages, for instance having a limited reach and requiring many resources (Salazar et al., 2014).

To address these issues, the use and evaluation of digital alternatives has recently come to the fore (Kleinsasser et al., 2015; Salazar et al., 2014). But, to our knowledge, none of the evaluated online sexual violence prevention programs have been particularly designed for female college students in India. To fill this research gap, we developed RISE-ON, an online sexual violence prevention program consisting of three modules, namely gender, sexual violence, and bystander education. RISE-ON was developed based on (a) the evaluation of the face-to-face prevention program RISE (Nieder et al., 2020), (b) evidence-based online sexual violence prevention programs, most importantly RealConsent (Salazar et al., 2014) and Take Care (Kleinsasser et al., 2015), and (c) state-of-the-art literature on the design of online programs (Clark & Mayer, 2016; Mayer, 2019). The evaluation of the three RISE-ON modules is the central aim of the present study.

### Sexual violence prevention – face-to-face programs

Since key risk factors for sexual violence include high levels of gender inequality and the acceptance of traditional gender roles (Hunnicutt, 2009; Weitzman, 2014), many positively evaluated face-to-face sexual violence prevention programs aim at promoting gender-equitable attitudes (Foshee et al., 2005; Taylor et al., 2013). For instance, the Safe Dates program by Foshee et al. (2005) focuses on changing norms associated with sexual violence and decreasing gender stereotyping. An evaluation study confirmed positive effects of the program (e.g., a decreased likelihood of experiencing sexual violence) mediated by a lower acceptance of traditional gender norms. Another focus of face-to-face programs is on sexual violence education, which includes describing the forms and risk factors of sexual violence as well as rape myths (Daigneault et al., 2015; Taylor et al., 2013). For instance, Daigneault et al. (2015) observed positive effects of a sexual violence awareness program in terms of increasing knowledge and improving attitudes (i.e., reduced acceptance of rape myths). One of the most recent prevention approaches in face-to-face programs focuses on bystander education, which describes how to intervene in potentially violent situations (Coker et al., 2011; Peterson et al., 2018). For instance, Peterson et al. (2018) compared the effectiveness of a bystander program with a traditional awareness program and found the bystander program to be more effective in reducing rape myth acceptance and increasing bystander efficacy and intervention.

The face-to-face sexual violence prevention program RISE, which was specifically designed for female college students in India, includes the topics of gender, sexual violence, and bystander education (Nieder et al., 2020). While the program produced no effects on sexual victimization and bystander behavior, an evaluation study clearly confirmed positive effects of the program concerning increases of knowledge (i.e., on gender, sexual violence, and bystander education) and changes of attitudes (i.e., increasing awareness of gender stereotypes, bystander efficacy, and intentions to intervene) up to six months later (Nieder et al., 2020). Thus, according to the transtheoretical model of behavior change (Prochaska & DiClemente, 1983), RISE provides a necessary foundation and important precondition for future behavior change. However, despite proven efficacy, RISE requires considerable resources (i.e., time, space, qualified trainers) and is limited in reach. Against this background, we developed RISE-ON, an online sexual violence prevention program that focuses -based on the evaluation of RISE- on changes of knowledge as well as attitudes. Thus, if RISE-ON proves to be efficacious in the present evaluation study, the online program has the potential to increase the effectiveness of sexual violence prevention programs in India by reducing costs, increasing implementation fidelity, and targeting larger populations (cf. Gottfredson et al., 2015).

### Sexual violence prevention – online programs

As face-to-face prevention programs require many resources (i.e., time, space, qualified trainers) and reach only a small number of people (Salazar et al., 2014), current research is increasingly focusing on online formats. Online programs have further advantages as well: The privacy of online environments can produce greater disclosure of sensitive topics, such as health-risk and sexual behaviors as well as violence (Clark-Gordon et al., 2019; Turner et al., 1998). Previous studies have also demonstrated that online learning is as effective as face-to-face learning (Neuhauser, 2002), and, in some contexts (e.g., the training of clinicians), can result in greater increases of knowledge (Dimeff et al., 2015).

Positive effects have also been found via evaluation studies of two online sexual violence prevention programs. Salazar et al. (2014) evaluated RealConsent*,* an online bystander program for male college students, and results showed that the program increased bystander behavior and decreased sexual violence perpetration. Participants in the intervention group also showed significant increases of knowledge on sexual violence, less adherence to traditional gender roles, less rape myth acceptance, as well as greater intentions to intervene as a bystander (Salazar et al., 2014). An evaluation of the online bystander intervention program Take Care by Kleinsasser et al. (2015) showed similar results. Students who participated in the program (80.6 % females) showed significantly greater bystander efficacy and relatively more bystander behavior vis-à-vis friends (but not strangers and acquaintances). Although online programs have shown promising effects, using online formats comes with challenges (Summers et al., 2005). Thus, the design of online programs (e.g., structure, interaction, and feedback) appears to be crucial for avoiding negative emotions and supporting students’ performance and motivation (Kauffman, 2015). Following this, we developed the online sexual violence prevention program RISE-ON.

### RISE-ON – an online sexual violence prevention program

Due to the lack of culture-sensitive online sexual violence prevention programs for female college students in India, we developed the three RISE-ON modules (i.e., Gender, Sexual Violence and Bystander) based on the evaluation of the face-to-face sexual violence prevention program RISE (Nieder et al., 2020) as well as on evidence-based online sexual violence prevention programs like RealConsent (Salazar et al., 2014) and Take Care (Kleinsasser et al., 2015). RISE originally included a fourth module (i.e., Healthy Relationships and Communication), but because the communication training (i.e., the key element of the fourth module) appeared less suitable for an online format, we decided to drop this module when developing RISE-ON. The face-to-face program RISE focused on women (Nieder et al., 2020), and previous research has recommended gender-specific prevention programs (Kumpfer et al., 2008); thus, RISE-ON similarly focuses on Indian female college students.

In the RISE-ON program, the Gender module (i.e., module G) focuses on gender-related knowledge (e.g., the difference between sex and gender) and attitudes (e.g., awareness of gender role stereotypes). The Sexual Violence module (i.e., module S) deals with knowledge on sexual violence (e.g., forms, prevalence, and risk factors of sexual violence). Because sex education is lacking in India (Brahme et al., 2020) and college students are exposed to many rape myths (Qureshi et al., 2020), this module focuses on the basic concept of consent and reducing the acceptance of rape myths. Finally, the Bystander module (i.e., module B) focuses on knowledge (e.g., bystander opportunities and intervention strategies) as well as attitudes (i.e., bystander efficacy and intentions to intervene).

As mentioned above, to have an effective online program is challenging (Summers et al., 2005) and requires an adequate design (Kaufman et al., 2019). Thus, to develop RISE-ON, we included six key design features. First, to reduce extraneous processing, we highlighted relevant material in each module on digital post-its. Second, to support the management of essential processing, we divided sections of the RISE-ON modules into learner-paced parts; this means we included a button click to allow students to decide individually when to continue with the program. Third, to foster generative processing, we used conversational language throughout all RISE-ON modules (Mayer, 2019). Next, since online learning requires a considerable amount of self-direction, the fourth design feature was to use a pedagogical agent (i.e., an animated (2D/3D) character or human instructor) to guide students through the online program (Clark & Mayer, 2016; Shiban et al., 2015). For the three RISE-ON modules, we included videos of a young Indian woman (i.e., a human instructor), because the presence of a human instructor was found to have positive effects on students’ visual attention, their recall of information, their perceived learning success, and their satisfaction (Wang & Antonenko, 2017). Additionally, by choosing a young Indian woman, we assured familiarity and relatedness with the agent (Shiban et al., 2015). Fifth, to further support the students’ motivation and satisfaction, we included feedback after the completion of each task (Hattie & Timperley, 2007; Li et al., 2020) Finally, the sixth design feature was to use gamification, meaning that we included gaming elements and techniques in online learning (Bovermann & Bastiaens, 2020; Tsay et al., 2018). Because students’ motivation and desire to engage increases when they can earn badges (Hakulinen et al., 2015), we awarded badges at the end of each RISE-ON module.

### Evaluation model and hypotheses

To evaluate RISE-ON, outcomes were – following the evaluation model of Kirkpatrick and Kirkpatrick (2006) – assessed on two levels, namely reaction and learning, with the latter including both knowledge and attitudes. Concerning the first level (i.e., students’ reaction to the program), we expect a high satisfaction with the three RISE-ON modules. Concerning the second level (i.e., learning), we tested the following specific hypotheses on knowledge and attitudes (including awareness and behavioral intentions):

1. Module Gender - Participants in the gender module will show an increase in gender-related knowledge as well as gender awareness compared to control group participants.
2. Module Sexual Violence *-* Participants in the sexual violence module will show an increase in sexual violence-related knowledge as well as positive attitudes toward sexual violence (i.e., less rape myth acceptance) compared to participants in the control condition.
3. Module Bystander - Participants in the bystander module will show an increase in bystander-related knowledge, bystander efficacy, and intentions to intervene as a bystander compared to participants in the control condition.

## Method

### Design of the study

For the current study, we used a pretest (T1)-posttest (T2) design and randomly assigned students from nine different classes (1st, 2nd and 3rd year mathematics and 2nd year commerce; class sizes varied between 21 and 36 students) to 1 of 3 groups, with each group composed of two out of three modules, namely (1) Gender & Sexual Violence (GS), (2) Gender & Bystander (GB), or (3) Sexual Violence & Bystander (SB). As every group included only two of the three modules, they each function as a treatment group for the two modules that were included in that group and as a control condition for the third (missing) module. For instance, the specific effects of the Gender module G can be tested by comparing the change between pre- and posttest in the module-specific outcomes between the conditions that included module G (i.e., group (1) and (2)) to the condition that did not include module G (i.e., group (3)). The sample of the present study was selected based on an existing cooperation with a college in New-Delhi that already took part in a previous study (i.e., the evaluation of the face-to-face prevention program RISE; Nieder et al., 2020). All classes of the English-language degree programs that had not taken part in the face-to-face prevention program RISE participated in the online program RISE-ON.

### Participants

Because we were interested in meaningful effects (i.e., the smallest significant effect sizes found in the evaluation study of RISE (Nieder et al., 2020) with *f*^2^ > 0.13), sample size was calculated based on a corresponding power analysis with GPower (i.e., test family: *F* test; statistical test: ANOVA, repeated measures, within-between interaction) and the following input parameters: *f*^2^ > 0.13, *α* = 0.05, power (= 1 − *α*) = 0.95, number of groups = 3, number of measurements = 2, correlation among repeated measures = 0.5, nonsphericity correction *ε* = 1. The result indicated that a total sample size of *N* = 234 would be sufficient to detect corresponding effects. To account for dropouts, we included a total of *N* = 252 students. Data from eight students had to be excluded (e.g., because of technical issues) so that the final sample consisted of *N* = 244 (GS: *N* = 82; GB: *N* = 81; SB: *N* = 81) female college students between the ages of 17 and 21 years (*M* = 18.87, *SD* = 0.92; GS: *M* = 18.79, *SD* = 0.87; GB: *M* = 19.09, *SD* = 0.95; SB: *M* = 18.74, *SD* = 0.92).

All participants came from middle- and upper-middle-class families and studied at the same college in New Delhi. One participant was married, 16.7 % of participants (GS: 20.7 %; GB: 14.8 %; SB: 14.8 %) indicated to be in a relationship and 82.9 % of participants (GS: 79.3 %; GB: 84.0 %; SB: 85.2 %) declared they were single. Furthermore, 20.0 % of participants (GS: 22.0 %; GB: 19.8 %; SB: 18.5 %) stated that they had been in a romantic relationship before. All 244 participants were Indian nationals, and the majority (95.1 %) was Hindu by religion (GS: 95.1 %; GB: 92.6 %; SB: 97.5 %). Almost all students (98.0 %) mentioned Hindi as their native language. However, because all study participants were following the English track at college, they were fluent in English as well.

At the time of pretest (T1), the three groups (GS, GB, SB) did not differ significantly regarding their relationship status, *χ²*(4) = 3.34, *p* = .503, relationship experience, *χ²*(2) = 0.31, *p* = .858, and religion, *χ²*(6) = 6.74, *p* = .346. Furthermore, although there was a significant age difference, *F*(2, 243) = 3.37, *p* = .036, η² = 0.027, none of the post-hoc *t*-tests with Bonferroni correction reached significance, where the smallest *p* = .051 for group 2 (GB) vs. group 3 (SB), with participants of group 2 being, on average, three months older.

### Data collection

Data were collected using LabVanced, an online platform for online studies, surveys, market research and e-learning developed by researchers in Osnabrück, Germany. Before starting with the online program, participants were informed about the study and asked for their consent to participate. Furthermore, participants received information about the college counseling service and the opportunity to seek help from the trainer in case of any stress during or after the program. To assure participants’ safety, to answer questions, and to assist them in case of technical problems, a trainer (i.e., an expert on sexual violence prevention) was present throughout the entire program.

In each of the three groups, two of the three modules (Gender, Sexual Violence and Bystander) were covered. At the beginning, participants completed an evaluation instrument (pretest) covering outcome measures on all three modules to assess their baseline scores. After that, they worked independently on two of the three modules, and, finally, they filled out the evaluation instrument again (posttest). The program was conducted in the English language within one session. Materials used in each of the three modules of the program were mainly videos of the human instructor (who guided students through the online program and provided information) as well as video- or text-based case studies of specific everyday situations that young Indian women may face (e.g., regarding different forms of sexual violence, consent or concrete bystander intervention strategies) and information slides (e.g., regarding key concepts and definitions). Online activities included mainly quizzes (e.g., answering multiple choice or yes/no questions after watching a video or reading a case study), open answer formats (e.g., responding to self-reflective questions) and other learning exercises (e.g., sorting facts or putting information in a correct order). Since participants determined their pace of work individually, the duration varied between 55 and 145 min.

### Outcome measures

Outcomes were measured on two levels (Kirkpatrick & Kirkpatrick, 2006), namely reaction and learning (i.e., knowledge and attitudes). All scales were adapted from the evaluation study of RISE (Nieder et al., 2020) and further refined for the current study. Outcomes on knowledge were divided into two parts, namely objective (i.e., assessed by multiple choice items with one correct answer) and subjective knowledge (i.e., assessed by participants’ own evaluation of knowledge). Measures on subjective knowledge and attitudes (i.e., gender awareness, sexual violence attitudes, bystander efficacy, and bystander intention) were measured on 6-point Likert-type subscales ranging from 1 (‘not agree at all’) to 6 (‘strongly agree’). All scales were coded so that higher scores represent preferable outcomes.

### Reaction

We used two items (‘I am satisfied with the online program’, ‘The online program was helpful’) to assess students’ reaction to the online prevention program.

### Gender module

To measure students’ objective knowledge about gender (*Gender Objective Knowledge*), we used three multiple-choice items with four options each (e.g., ‘The main sources of the development of gender roles are: (a) biological characteristics that define people as female or male, (b) specific beliefs, expectations, and attitudes, (c) peoples’ personality, (d) none of them’). Students’ subjective knowledge about gender (*Gender Subjective Knowledge*) was assessed by three items (e.g., ‘I know exactly what makes sex and gender different’). Cronbach's alpha was α_T1_ = 0.72 and α_T2_ = 0.85. To measure students’ awareness of gender stereotypes (*Gender Awareness*), we used three items (e.g., ‘I recognize when other people use gender stereotypes’). Cronbach's was α_T1_ = 0.61 and α_T2_ = 0.72.

### Sexual violence module

To measure students’ objective knowledge about sexual violence (*Sexual Violence Objective Knowledge*), we used four multiple-choice items with four options each (e.g., ‘Victim blaming: (a) does not lead to self-blame of the victim, (b) is emphasized through rape myths, (c) does not happen in court, (d) is in some situations a correct response’). Students’ subjective knowledge about sexual violence (*Sexual Violence Subjective Knowledge*) was assessed by three items (e.g., ‘I know exactly what rape myths and victim blaming are’). Cronbach's alpha was α_T1_ = 0.79 and α_T2_ = 0.83. The subscale *Sexual Violence Attitudes* was adapted from the Attitudes Toward Rape Victims Scale (Ward, 1988). Students rated nine statements about attitudes toward rape victims (e.g., ‘A raped person is usually an innocent victim’). Cronbach's alpha was α_T1_ = 0.62 and α_T2_ = 0.65.

### Bystander module

To measure students’ objective knowledge about bystander education (*Bystander Objective Knowledge*), we used four multiple-choice items with four options each (e.g., ‘As a bystander: (a) One can intervene most effectively when the situation is over, (b) one should always intervene regardless of the circumstances, (c) one should primarily address the perpetrator, (d) one can make the situation worse for the victim). Students’ subjective knowledge about bystander education (*Bystander Subjective Knowledge*) was measured by four items (e.g., ‘I know strategies for how to behave as a bystander in situations of sexual violence’). Cronbach's alpha was α_T1_ = 0.85 and α_T2_ = 0.91. The subscale *Bystander Efficacy* was adapted from the Slaby Bystander Efficacy Scale (Slaby et al., 1994) and the Mentors in Violence Program Efficacy Scale (Cissner, 2009). Students rated nine statements about attitudes toward bystander efficacy (e.g., ‘I can learn to do or say the kinds of things that help prevent violence in my community’). Cronbach's alpha was α_T1_ = 0.71 and α_T2_ = 0.74. Students’ intention to intervene as a bystander (*Bystander Intention)* was assessed by four items (e.g., ‘I want to step in if somebody is being harassed’). Cronbach's alpha was α_T1_ = 0.77 and α_T2_ = 0.84.

### Statistical analyses

For statistical analyses, we used SPSS Version 27. Preliminary data analyses covered descriptive statistical analyses and pretest differences between groups. To evaluate the short-term effects of the three RISE-ON modules, we performed mixed ANOVAs with time as a within-subjects factor (pretest and posttest) and group (GS, GB, SB) as a between-subjects factor. For post-hoc tests, we used a series of *t*-tests with Bonferroni correction. In case of a significant Group × Time interaction, we performed one-factorial (Group: GS vs. GB vs. SB) ANOVAs on the change score (posttest minus pretest) and tested the specific contrast comparing the two intervention conditions with the control condition of each module.

## Results

### Preliminary analyses

We found no significant group differences at T1, except for *Sexual Violence Objective Knowledge, F*(2, 243) = 3.03, *p* = .050, η² = 0.025. Thus, students in the third group (SB) had significantly lower baseline scores (*p* = .048) than students in the first group (GS; see Table 1 for details). As mentioned above, there was a significant age difference between groups. Since post-hoc *t*-tests reached, at most, marginal significance and there was no conceptual reason that an age difference of three months would affect the effectiveness of the modules in any way, we dropped age as a covariate in the final analyses. Finally, there were no missing values due to the design of the online program, which required answers for each item.

**Table 1:** Means and Standard Deviations for Outcome Variables of the RISE-ON Modules.

	Group 1: Gender & Sexual violence (*n* = 82)		Group 2: Gender & Bystander (*n* = 81)		Group 3: Sexual violence & Bystander (*n* = 81)	
	T1	T2	T1	T2	T1	T2
	*M* (*SD*)	*M* (*SD*)	*M* (*SD*)	*M* (*SD*)	*M* (*SD*)	*M* (*SD*)
Reaction	–	5.72 (0.49)	–	5.66 (0.62)	–	5.72 (0.62)
Gender						
Awareness	3.74 (1.27)	5.00 (1.05)	3.76 (1.17)	4.93 (1.10)	3.77 (1.12)	4.70 (1.09)
Knowledge (objective)	0.47 (0.29)	0.78 (0.29)	0.44 (0.28)	0.63 (0.28)	0.41 (0.28)	0.45 (0.29)
Knowledge (subjective)	3.44 (1.36)	5.54 (0.64)	3.37 (1.09)	5.23 (0.98)	3.38 (1.29)	5.00 (0.93)
Sexual Violence						
Attitudes	4.71 (0.72)	4.91 (0.77)	4.48 (0.90)	4.48 (0.82)	4.69 (0.70)	4.87 (0.79)
Knowledge (objective)	0.42 (0.28)	0.66 (0.27)	0.39 (0.31)	0.48 (0.30)	0.31 (0.26)	0.60 (0.28)
Knowledge (subjective)	3.42 (1.28)	5.59 (0.53)	3.52 (1.19)	5.00 (1.00)	3.51 (1.10)	5.56 (0.73)
Bystander						
Efficacy	4.31 (0.80)	4.93 (0.69)	4.26 (0.84)	4.72 (0.76)	4.37 (0.66)	4.96 (0.71)
Knowledge (objective)	0.43 (0.22)	0.36 (0.26)	0.43 (0.23)	0.42 (0.26)	0.42 (0.25)	0.39 (0.20)
Knowledge (subjective)	3.39 (1.16)	4.60 (1.23)	3.36 (1.27)	5.28 (1.01)	3.31 (1.12)	5.57 (0.68)
Intention	4.89 (0.93)	5.47 (0.73)	4.85 (1.10)	5.20 (0.95)	4.97 (0.92)	5.45 (0.74)

*Note.* T1 = Pretest, T2 = Posttest. Scores range from 1 to 6. Exception: All Knowledge (Objective) Scores range from 0 to 1.

### Reaction

Participants showed a high satisfaction score of *M* = 5.70 (*SD* = 0.47) across all groups, indicating high satisfaction with the modules of the online program (GS: *M* = 5.72, *SD* = 0.49; GB: *M* = 5.66, *SD* = 0.62; SB: *M* = 5.72, *SD* = 0.62).

### Gender module

**Fig. 1:**
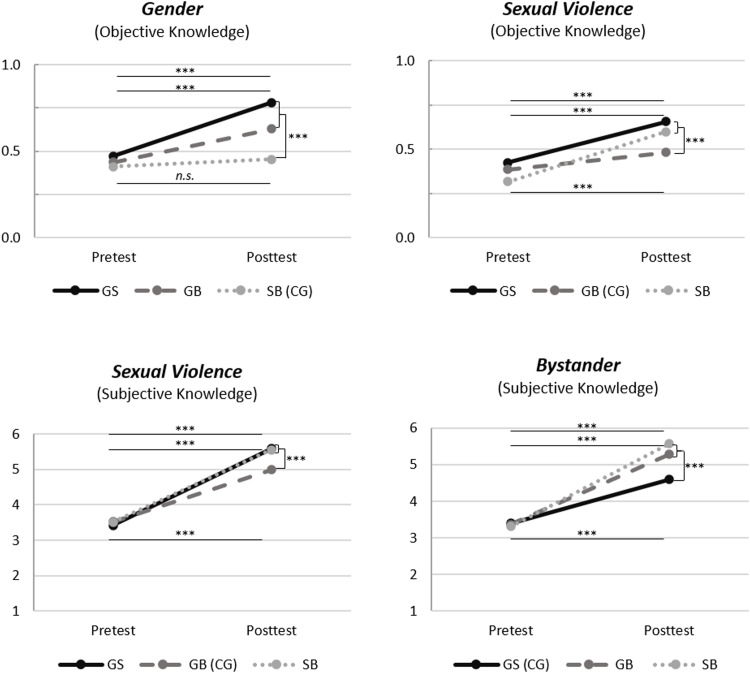
Group-specific effects of the three RISE-ON modules. *Note*. Horizontal lines represent pre-post differences per group (the order of lines per graph follows the order of groups at posttest). Vertical lines indicate results of the specific contrasts comparing the two intervention conditions with the control condition of each module. ^†^*p* < .10; * *p* < .05; ^⁎⁎^*p* < .01; ^⁎⁎⁎^*p* < .001.

Regarding the module about gender, we considered three different outcomes for knowledge (objective as well as subjective) and attitudes (i.e., awareness). There were significant main effects of Group and Time on *Gender Objective Knowledge* and significant main effects of Time but not Group on *Gender Subjective Knowledge* and *Gender Awareness* (see Table 1, Table 2 and Fig. 1 for details). Furthermore, there was a significant Group × Time interaction effect on *Gender Objective Knowledge* (medium effect, see Table 2 for η²) but not on *Gender Subjective Knowledge* and *Gender Awareness.*

**Table 2:** Mixed ANOVA Results for Outcome Variables of the RISE-ON Modules.

	Time				Group				Time × Group			
	F	df	p	η²	F	df	p	η²	F	df	p	η²
Gender												
Awareness	195.20	241	<0.000	.448	0.47	241	.625	.004	1.63	241	.199	.013
Knowledge (objective)	75.51	241	<0.000	.239	13.98	241	<0.000	.104	13.85	241	<0.000	.103
Knowledge (subjective)	482.96	241	<0.000	.667	2.61	241	.076	.021	2.79	241	.064	.023
Sexual Violence												
Attitudes	8.41	240	.004	.034	5.46	240	.005	.043	2.16	240	.117	.018
Knowledge (objective)	161.31	241	<0.000	.401	3.86	241	.022	.031	12.19	241	<0.000	.092
Knowledge (subjective)	610.21	241	<0.000	.717	2.84	241	.060	.023	7.78	241	.001	.061
Bystander												
Efficacy	127.92	240	<0.000	.348	1.61	240	.202	.013	1.04	240	.356	.009
Knowledge (objective)	3.69	240	.056	.015	0.50	240	.606	.004	1.28	240	.279	.011
Knowledge (subjective)	544.02	240	<0.000	.694	5.08	240	.007	.041	16.19	240	<0.000	.119
Intention	67.57	240	<0.000	.220	1.22	240	.298	.010	1.40	240	.249	.012

Regarding *Gender Objective Knowledge*, post-hoc *t*-tests showed significant increases in group 1 (GS) from T1 to T2 (*p* < .001) and in group 2 (GB) from T1 to T2 (*p* < .001), but not in the control condition (group 3 (SB); T1 to T2, *p* = .133). Additionally, there were significantly higher scores at T2 in group 1 (GS) as well as in group 2 (GB) compared to the control condition (*p*s < 0.001), and, unexpectedly, significantly higher scores at T2 in group 1 (GS) compared to group 2 (GB; *p* = .003). Contrast analyses further confirmed significantly higher change scores in group 1 and 2 compared to the control condition (group 3; *p* < .001).

Overall, there were similar significant increases of subjective knowledge and awareness in all three groups, and, in the case of objective knowledge, these increases were specific to those groups that contained the Gender module.

### Sexual violence module

Regarding the module about sexual violence, we considered three different outcomes for knowledge (objective as well as subjective) and attitudes. There were significant main effects of Group and Time on two scales, namely *Sexual Violence Objective Knowledge* and *Sexual Violence Attitudes* and a significant main effect of Time but not Group on *Sexual Violence Subjective Knowledge* (see Table 1, Table 2 and Fig. 1 for details). Furthermore, there were significant Group × Time interaction effects on *Sexual Violence Objective Knowledge* (medium effect, see Table 2 for η²) and *Sexual Violence Subjective Knowledge* (medium effect) but not *Sexual Violence Attitudes.*

Concerning *Sexual Violence Objective Knowledge*, post-hoc *t*-tests revealed significant increases in all three groups from T1 to T2 (*p*s < 0.001). Additionally, there were significantly higher scores at T2 in group 1 (GS) and in group 3 (SB) compared to the control condition (group 2 (GB); *p* < .001 and *p* = .021, respectively) and, also as expected, no significant differences at T2 between group 1 (GS) and group 3 (SB; *p* = .678). Contrast analyses further confirmed significantly higher change scores in group 1 and 3 compared to the control condition (group 2; *p* < .001).

For *Sexual Violence Subjective Knowledge*, post-hoc *t*-tests indicated significant increases in all three groups from T1 to T2 (*p*s < 0.001). Additionally, we found significantly higher scores at T2 in group 1 (GS) and in group 3 (SB) compared to the control condition (group 2 (GB); *p*s < 0.001) but no differences at T2 between group 1 (GS) and group 3 (SB; *p* = 1.000). Contrast analyses further confirmed significantly higher change scores in group 1 and 3 compared to the control condition (group 2; *p* < .001).

Overall, there was an increase for all outcomes across groups (except for *Sexual Violence Attitudes* in the control condition) that was, in the case of knowledge, particularly pronounced in the groups that contained the sexual violence module.

### Bystander module

Regarding the module about bystander education, we considered four different outcomes for knowledge (objective as well as subjective), attitudes, and intention. There were significant main effects of Group and Time on *Bystander Subjective Knowledge* and significant main effects of Time but not Group on *Bystander Objective Knowledge, Bystander Efficacy* and *Bystander Intention* (see Table 1, Table 2 and Fig. 1 for details). Furthermore, there was a significant Group × Time interaction effect on *Bystander Subjective Knowledge* (medium effect, see Table 2 for η²) but not *Bystander Objective Knowledge, Bystander Efficacy* and *Bystander Intention.*

Regarding *Bystander Subjective Knowledge*, post-hoc *t*-tests showed significant increases in all three groups from T1 to T2 (*p*s < 0.001). Additionally, we found significantly higher scores at T2 in group 2 (GB) and in group 3 (SB) compared to the control condition (group 1 (GS); *p*s < 0.001) but no significant differences at T2 between group 2 (GB) and group 3 (SB; *p* = .151). Contrast analyses further confirmed significantly higher change scores in group 2 and 3 compared to the control condition (group 1; *p* < .001).

Overall, there were similar significant increases of bystander attitudes (i.e., efficacy and intention) in all three groups, and, in the case of subjective knowledge, these increases were specific to those groups that contained the Bystander module.

## Discussion

In the present study, we evaluated the short-term effects of the three RISE-ON modules of Gender, Sexual Violence, and Bystander behavior. As expected, students in all three groups showed high satisfaction with the program, thus indicating positive reactions to all three modules. Furthermore, we found significant and module-specific effects of the three modules in terms of increasing students’ knowledge. Thus, students who participated in the Gender module showed significant increases of objectively assessed knowledge compared to students in the control condition. Similarly, students who took part in the Sexual Violence module showed both significant increases of objectively and subjectively assessed knowledge compared to students in the control condition. Finally, students who participated in the Bystander module rated their knowledge on bystander education significantly higher than students in the control condition. Thus, four of our six hypotheses regarding changes of knowledge were supported, which goes along with results of other evaluation studies of face-to-face (Daigneault et al., 2015) and online programs (Salazar et al., 2014). In comparison to the face-to-face program RISE (Nieder et al., 2020), RISE-ON was slightly less effective in terms of imparting knowledge on sexual violence and bystander education (despite a medium effect size), but even more effective than RISE regarding gender-related knowledge.

However, different from the above-mentioned evaluation studies (Coker et al., 2011; Daigneault et al., 2015; Foshee et al., 2005; Nieder et al., 2020; Salazar et al., 2014), we found no module-specific differences between groups regarding changes of attitudes, namely increased awareness of gender stereotypes (after module G), reduced rape myth acceptance (after module S), and improved bystander efficacy and intentions to intervene (after module B). Thus, none of our four hypotheses regarding changes of attitudes were confirmed by module-specific increases. Rather, what we found was a general effect independent of the specific combination of modules. More specifically, gender awareness and bystander attitudes scores increased in all three groups, that is, across all combinations of the three modules.

This could be interpreted in either of two ways. First, one could take this as evidence for ineffectiveness (since there is no module-specific effect), which could be due to several reasons: One explanation for the non-significant findings could have to do with the length of the program, as it included only one session and, thus, was too short to affect participants’ attitudes. Additionally, the online format of the program may explain why we found significant increases of knowledge but no change of attitudes, as online formats are known to be particularly suitable for imparting theoretical knowledge (Dimeff et al., 2015). Consequently, since the expression of emotions plays a crucial part in attitude change (van Kleef et al., 2015), the content of the RISE-ON modules might have been too impersonal or not interactive enough and, thus, less effective in terms of changing attitudes.

Second, one could interpret this general effect as a spillover effect, meaning that all modules (despite their actual focus) had an impact on students’ attitudes associated – in one way or another – with the more global issue of sexual violence. For example, students who participated in the modules on Gender and Sexual Violence but not in the module on Bystander may have shown an increase of intentions to intervene as a bystander after learning about gender inequalities in India and negative consequences of sexual violence. Similarly, students who participated in the modules on Sexual Violence and Bystander but not in the module on Gender may have shown increasing awareness of gender stereotypes after learning about the high prevalence of sexual violence against women compared to men. To judge between these two alternative interpretations and to make a reliable statement regarding the effectiveness of the three modules on attitudes, future research needs to compare outcomes of each module or combinations thereof with a placebo or waitlist control group.

In summary, we were able to show that the three RISE-ON modules are effective in terms of increasing participants’ knowledge on gender, sexual violence, and bystander education. Concerning attitude change, there was an overall improvement independent of the specific combination of the three RISE-ON modules. Thus, RISE-ON represents a promising starting point for online sexual violence prevention programs in India. However, the impact of the program on modifying attitudes and behavior might be limited and needs to be addressed in future research.

### Study strengths and limitations

Important strengths of the current study concern the culture-sensitive development of RISE-ON, which also characterizes the face-to-face prevention program RISE (Nieder et al., 2020) from which it was derived. We also considered several effective design features for the program development, for instance, using a human instructor (Wang & Antonenko, 2017), implementing regular feedback (Hattie & Timperley, 2007), and gamification elements (Bovermann & Bastiaens, 2020). As sexual violence is a sensitive topic, we assured students’ safety and well-being during and after participation in the online program by making them aware of counseling services and support structures on the college campus as well as ensuring the presence of a professional trainer.

A key strength of RISE-ON is its potential for widespread dissemination. As the present evaluation study has demonstrated the efficacy of the three RISE-ON modules, an important next step would be to perform effectiveness studies (Gottfredson et al., 2015). As the online format of RISE-ON guarantees perfect implementation fidelity and is a cost-effective way to promote sexual violence prevention in India, an important question for future research would be how the program performs in less standardized settings (such as, for example, when it is freely available at any time and in any context).

However, there are limitations of the present study as well. Due to the specific study design, it seems plausible but remains an open question whether RISE-ON leads to a change in attitudes. To address this questions, future studies should include an alternative treatment or waitlist control group design. A further limitation concerns the scales used to evaluate the three RISE-ON modules. Although 8 out of 10 scales show approved reliability in terms of internal consistency, the low Cronbach's alpha of two subscales, namely *Gender Awareness* (i.e., αs = 0.61 at pretest) and *Sexual Violence Attitudes* (i.e., αs = 0.62 at pretest and i.e., αs = 0.65 at posttest) is of concern, and these results should be interpreted with caution. Finally, since the online program was specifically designed for female college students in India, we cannot generalize our results to other Indian sub-samples (e.g., women from other socio-economic backgrounds). Additionally, due to the huge diversity in India, results might not generalize to female college students in other regions of the country. Since the sample of the present study was comparatively small, results may not me generalized and should be interpreted with caution.

### Implications for future research

Although increases of knowledge and potentially attitudes represent an important first step toward preventing sexual violence against women in India, they are not sufficient to achieve a long-lasting impact. Thus, future online sexual violence prevention programs should increasingly focus on positive changes in behavior and aim at measuring long-term effects. Moreover, as sexual violence does not only affect female college students but affects women from all socio-economic backgrounds, it is important for future sexual violence prevention programs to focus on women from all parts of society. Thus, future research should focus on testing generalizability of sexual violence prevention programs and providing programs in local languages. Finally, because it takes a whole community to achieve long-lasting changes in terms of sexual violence prevention, it is crucial to focus increasingly on sexual violence prevention programs aimed at men. In this context, the use of online programs represents a promising approach, especially given their large reach and easy distribution.

## Declaration of competing interest

The authors declare that they have no known competing financial interests or personal relationships that could have appeared to influence the work reported in this paper.
